# Early pharmacologic venous thromboembolism prophylaxis and in-hospital mortality in ICU patients with intracerebral hemorrhage: a landmark, propensity-weighted cohort study

**DOI:** 10.3389/fneur.2026.1849021

**Published:** 2026-09-03

**Authors:** Hongfu Chen, Jianyong Ji, Hui Zhang, Er Nie, Qing Lan

**Affiliations:** 1 Department of Neurosurgery, The Affiliated Hospital of Xuzhou Medical University, Xuzhou, Jiangsu, China; 2 Department of Neurosurgery, The Second Affiliated Hospital of Soochow University, Suzhou, Jiangsu, China; 3 Department of Neurosurgery, Liaocheng People’s Hospital, Liaocheng, Shandong, China

**Keywords:** critical care, in-hospital mortality, intracerebral hemorrhage, landmark analysis, thromboprophylaxis, venous thromboembolism

## Abstract

**Background and purpose:**

The optimal timing of pharmacologic venous thromboembolism (VTE) prophylaxis in intracerebral hemorrhage (ICH) is uncertain. Using a landmark design that classifies exposure only by information available at a fixed time point, we evaluated whether initiation of pharmacologic VTE prophylaxis within 48 h of ICU admission is associated with in-hospital mortality.

**Methods:**

Retrospective cohort study in the Medical Information Mart for Intensive Care IV (MIMIC-IV v3.1). Adults with non-traumatic ICH admitted to the ICU (2008–2019) who were alive and in hospital at a 48-h landmark were included. Exposure was fixed at the landmark: early prophylaxis = pharmacologic prophylaxis started ≤48 h; no early prophylaxis = not started by 48 h (comprising later [delayed] initiators and never-treated patients). Time zero and eligibility were ICU admission + 48 h. Inverse probability of treatment weighting (IPTW) adjusted for 17 baseline covariates including the baseline Glasgow Coma Scale (GCS). The primary outcome was in-hospital mortality (a binary outcome, with discharge alive as the competing event); the secondary outcome was coded VTE (binary). Missing GCS was handled by multiple imputation.

**Results:**

Of 2,754 ICH ICU patients, 2,407 reached the 48-h landmark and 2,380 formed the analytic cohort (early prophylaxis, *n* = 655; no early prophylaxis, *n* = 1,725) after excluding 27 receiving therapeutic anticoagulation within 48 h. In-hospital death occurred in 371 patients (early prophylaxis 10.8%, no early prophylaxis 17.4%). After IPTW (17/17 covariates standardized mean difference <0.1), early prophylaxis was associated with lower in-hospital mortality (adjusted odds ratio [OR] 0.72, 95% confidence interval [CI] 0.55–0.94; weighted risk difference −4.0%; cause-specific hazard ratio 0.65, 95% CI 0.50–0.86, *p* = 0.003). There was no statistically significant difference in coded VTE (adjusted OR 1.20, 95% CI 0.85–1.69; *p* = 0.31). The association was directionally consistent across most sensitivity analyses—including a model that treated prophylaxis initiation as a time-varying exposure to reduce immortal-time bias (HR 0.61, 95% CI 0.49–0.76) and a competing-risk cumulative-incidence analysis—although the 24-h landmark estimate was not statistically significant.

**Conclusion:**

In this landmark cohort, initiation of pharmacologic VTE prophylaxis within 48 h was associated with lower in-hospital mortality without a statistically significant difference in coded VTE. Because key neuroimaging severity measures (hematoma volume, location, intraventricular extension, and expansion) were unavailable, residual confounding by indication cannot be excluded and a causal interpretation is not warranted. Randomized trials are needed.

## Introduction

Intracerebral hemorrhage (ICH) accounts for 10–15% of strokes and carries a 1-year mortality exceeding 40% (1). Critically ill patients with ICH face high risks of both thromboembolic and hemorrhagic complications; venous thromboembolism (VTE) occurs in 2–13% of patients and increases morbidity and mortality (2–4). Guidelines differ on the timing of pharmacologic prophylaxis: the 2022 AHA/ASA guideline indicates that low-dose unfractionated or low-molecular-weight heparin may be reasonable to start 24–48 h after ICH onset in selected patients (1), and the Neurocritical Care Society and European Stroke Organization guidelines similarly endorse pharmacologic prophylaxis in the early days after ICH once bleeding has ceased, although their specific timing thresholds differ (5, 6). This variation reflects a theoretical concern that early anticoagulation might promote hematoma expansion, balanced against the thrombotic risk of delay.

Studies evaluating pharmacologic thromboprophylaxis after spontaneous ICH—including observational cohorts, a randomized trial, and several systematic reviews and meta-analyses—have generally suggested that initiation after radiographic stabilization may not substantially increase hematoma expansion, although the definitions of “early” treatment, patient selection, and adjustment for neurological severity have varied considerably (7–13). Much of this evidence derives from retrospective cohorts and aggregate meta-analyses with limited control for treatment-selection bias and treatment timing, and these uncertainties have contributed to variation in guideline recommendations and clinical practice.

Prior observational studies have additionally been limited by immortal-time bias, confounding by indication, and incomplete adjustment for neurological severity (14–16). Immortal-time bias arises when patients must survive long enough to receive an intervention; confounding by indication arises when patients with more severe presentations are treated differently. We therefore used a landmark design that classifies exposure at 48 h using only information available by then, combined with IPTW that includes a baseline neurological severity measure (GCS). Our objective was to estimate the association between initiation of pharmacologic VTE prophylaxis within 48 h of ICU admission and in-hospital mortality, framed as association rather than cause.

## Methods

### Study design and data source

Retrospective cohort study using MIMIC-IV v3.1 (17), a de-identified database from the Beth Israel Deaconess Medical Center (2008–2019). The database was approved by the institutional review boards of the Massachusetts Institute of Technology and Beth Israel Deaconess Medical Center, with waiver of individual consent. Reporting follows STROBE (18).

### Study population

We included adults (≥18 years) with a primary diagnosis of non-traumatic ICH (ICD-9431.x; ICD-10 I61.x) admitted to an ICU, taking one first ICU stay per hospital admission. We excluded traumatic ICH, subarachnoid and subdural hemorrhage, and, for the landmark analysis, patients not alive and in hospital at 48 h after ICU admission. Patients receiving therapeutic-dose anticoagulation within 48 h were excluded from the analytic cohort (see Exposure).

### Landmark design and exposure

To avoid immortal-time bias, exposure was classified at a 48-h landmark (19) using only information available by 48 h. Early prophylaxis was pharmacologic VTE prophylaxis (unfractionated heparin [UFH] 5,000 units subcutaneously, or enoxaparin 30–40 mg subcutaneously) with the first dose administered ≤48 h after ICU admission, ascertained from the electronic medication administration record. No early prophylaxis denotes patients not started by 48 h; this group comprises patients who initiated prophylaxis later (delayed) and patients never treated during the admission. Initiation of prophylaxis after the 48-h landmark did not change this baseline classification. Eligibility and time zero were ICU admission + 48 h. Therapeutic-dose anticoagulation (heparin infusion, intravenous heparin, or treatment-dose low-molecular-weight heparin) begun within 48 h was identified separately and excluded; therapeutic anticoagulation begun after 48 h did not trigger this exclusion.

### Outcomes

The primary outcome was in-hospital mortality. The secondary outcome was coded VTE (deep vein thrombosis or pulmonary embolism; ICD-9453.x, 415.1x; ICD-10 I80.x, I82.x, I26.x), analyzed as a binary in-hospital outcome. Because administrative codes do not provide a reliable VTE onset time and present-on-admission flags are unavailable in MIMIC-IV, a time-to-VTE analysis was not performed.

### Covariates and severity

The 17 baseline covariates were: age, sex, Charlson comorbidity components (myocardial infarction, congestive heart failure, peripheral vascular disease, cerebrovascular disease, dementia, chronic obstructive pulmonary disease, diabetes, renal disease, cancer), hypertension, atrial fibrillation, insurance (Medicare, Medicaid), emergency admission, and the baseline Glasgow Coma Scale (GCS) total as a severity measure. Because MIMIC-IV v3.1 contains no GCS-total field, total GCS was computed as the sum of the eye, verbal, and motor components recorded at the same assessment (identical charttime)—the earliest such synchronized assessment after ICU admission and before the earlier of first prophylaxis and the 48-h landmark. Components charted at different times were not combined into a total; 99.3% of GCS records had all three components co-charted, so a single-timepoint total was available for essentially all patients with any GCS. The verbal component is text-coded; for intubated patients it is recorded as “No Response–ETT” with a value of 1 (the standard MIMIC-IV convention), which we used as recorded—a numerically available score of 1 that does not reflect a directly testable verbal response—and flagged as intubation-affected; total GCS therefore remained obtainable in most intubated patients. Missing or unassessable components were never scored as zero; missing total GCS was handled by multiple imputation, and a motor-GCS model (motor is unaffected by intubation) was used as a sensitivity analysis. Neurosurgical procedures before the landmark (craniotomy or hematoma evacuation, and external ventricular drainage) were extracted but, because most procedures did not consistently precede the first prophylactic dose, were used for subgroup and interaction analysis rather than in the primary propensity model.

### Statistical analysis

Propensity scores were estimated by logistic regression on the 17 covariates and used to compute stabilized IPTW (20), truncated at the 1st/99th percentiles (21). Balance was assessed by absolute standardized mean differences (SMD; adequate balance defined as <0.1) (22). Because discharge alive is a competing event for in-hospital death, the primary mortality estimate was the weighted logistic odds ratio and risk difference, which directly estimate the probability of in-hospital death and do not censor discharge alive. As a supporting analysis we report a weighted cumulative-incidence (Aalen–Johansen) analysis that accounts for event timing with discharge alive as the competing event, and a weighted Cox cause-specific hazard ratio (time from the 48-h landmark, discharge alive censored; robust variance; proportional-hazards assessed) as a supplementary time-to-event estimate. VTE was modeled by weighted logistic regression with a weighted risk difference. Missing GCS (9% in the early prophylaxis group, <1% otherwise) was handled by multiple imputation (20 imputations), with estimates pooled by Rubin’s rules; complete-case and motor-GCS-substitution analyses were used as sensitivity analyses. Further sensitivity analyses were: an ICU length-of-stay ≥48-h landmark denominator; 24-h and 72-h landmarks; exclusion of all I62.x codes; overlap weighting; a doubly-robust weighted-and-adjusted model; a model that treated prophylaxis initiation as a time-varying exposure (exposure 0 before the actual first dose and 1 thereafter) to reduce immortal-time bias; and the E-value (23) for the point estimate and the confidence limit nearest the null. Subgroup analyses by pre-landmark neurosurgical management (surgical vs. conservative) included a treatment-by-subgroup interaction test. A three-group description (early/delayed/no prophylaxis) is reported for context only and is not an unbiased fixed-group effect comparison. Analyses used Python 3.13 (pandas, scikit-learn, statsmodels, lifelines); two-sided *α* = 0.05.

## Results

### Study population

**Figure 1:**
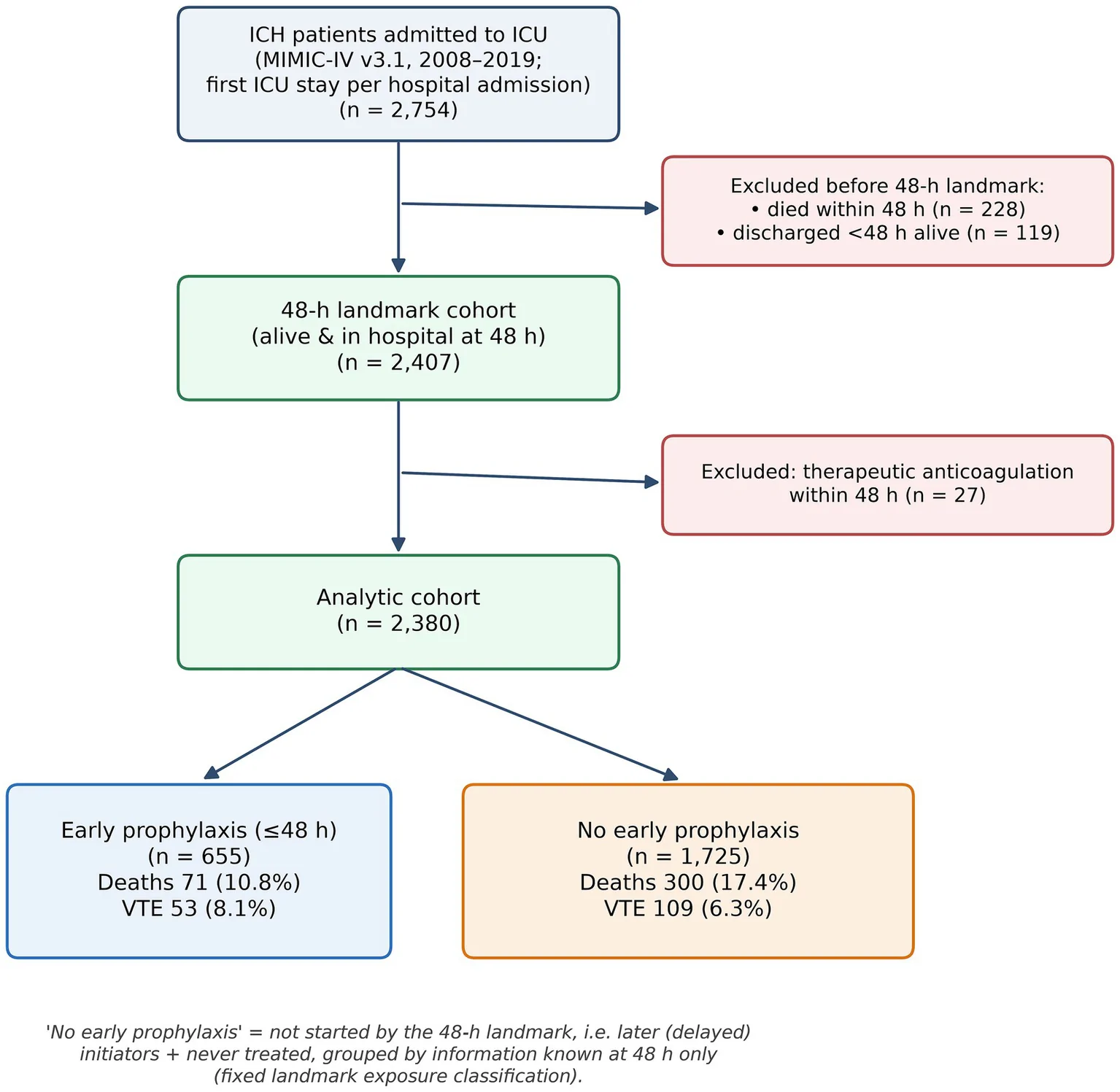
Study flow. From 2,754 ICH ICU first-stay patients, 2,407 were alive and in hospital at the 48-h landmark (Excluded: 228 died, 119 discharged alive within 48 h); after excluding 27 with therapeutic anticoagulation within 48 h, 2,380 formed the analytic cohort. Exposure was classified at 48 h using information available by then; “No early prophylaxis” comprises later (delayed) initiators and never-treated patients (fixed landmark exposure classification).

Of 2,754 ICH ICU first-stay patients, 2,407 were alive and in hospital at the 48-h landmark (347 excluded, 228 died and 119 were discharged alive within 48 h). After excluding 27 patients receiving therapeutic-dose anticoagulation within 48 h, the analytic cohort comprised 2,380 patients (early prophylaxis, *n* = 655; no early prophylaxis, *n* = 1,725; Figure 1). Among treated patients the first prophylactic dose occurred at a median of 45.4 h (IQR 26.8–69.7) after ICU admission, and the early exposure was predominantly UFH (early prophylaxis group: UFH 642, enoxaparin 13; 98.0% UFH), precluding a meaningful drug-agent subgroup analysis.

### Severity data and balance

**Figure 2:**
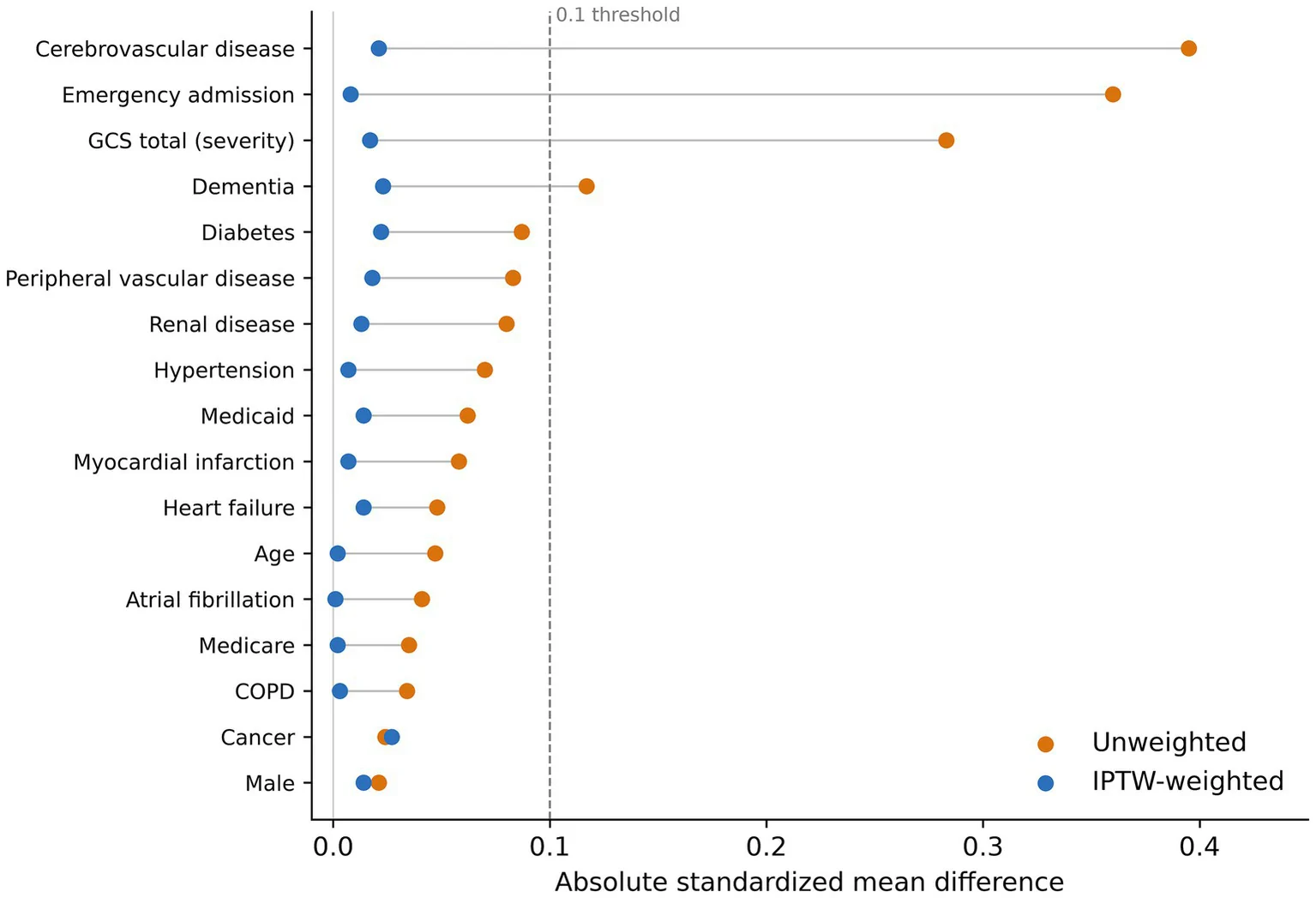
Covariate balance. Absolute standardized mean differences before and after IPTW for the 17 propensity-score covariates, including GCS total (severity). Values are from a representative imputed dataset; across all 20 imputations, 17/17 covariates achieved SMD < 0.1 (adequate balance).

Baseline GCS was available in 96.9% of patients (analytic cohort: early prophylaxis 91.0%, no early prophylaxis 99.8%); the verbal component was an ETT-coded score of 1 (numerically available but not a directly testable verbal response) in 34% of complete assessments, motivating a motor-GCS sensitivity model (see below). Before weighting, the groups differed most in cerebrovascular disease (SMD 0.40), emergency admission (0.36), and GCS total (0.28) (Table 1). After IPTW, all 17 covariates achieved SMD < 0.1 (adequate balance; Figure 2); and the propensity-score distributions showed adequate overlap (Supplementary Figure 1); the effective sample size was approximately 2,208across imputations and weights ranged 0.46–1.97.

**Table 1:** Baseline characteristics by exposure group at the 48-h landmark, before and after IPTW.

Characteristic	Early prophylaxis (*n* = 655)	No early prophylaxis (*n* = 1,725)	Unweighted SMD	Weighted SMD
Age, years (mean ± SD)	69.1 ± 14.9	68.3 ± 15.6	0.047	0.002
Male, *n* (%)	354 (54.0)	950 (55.1)	−0.021	−0.014
Baseline GCS total (mean ± SD)^*^	12.1 ± 3.5	11.0 ± 4.1	0.283	0.017
Myocardial infarction, *n* (%)	66 (10.1)	145 (8.4)	0.058	0.007
Congestive heart failure, *n* (%)	98 (15.0)	229 (13.3)	0.048	−0.014
Peripheral vascular disease, *n* (%)	25 (3.8)	96 (5.6)	−0.083	−0.018
Cerebrovascular disease, *n* (%)	259 (39.5)	374 (21.7)	0.395	0.021
Dementia, *n* (%)	58 (8.9)	100 (5.8)	0.117	0.023
COPD, *n* (%)	77 (11.8)	222 (12.9)	−0.034	−0.003
Diabetes, *n* (%)	190 (29.0)	434 (25.2)	0.087	0.022
Renal disease, *n* (%)	99 (15.1)	213 (12.3)	0.080	0.013
Cancer, *n* (%)	79 (12.1)	195 (11.3)	0.024	0.027
Hypertension, *n* (%)	534 (81.5)	1,358 (78.7)	0.070	0.007
Atrial fibrillation, *n* (%)	198 (30.2)	489 (28.3)	0.041	0.001
Emergency admission, *n* (%)	427 (65.2)	1,396 (80.9)	−0.360	−0.008
Medicare, *n* (%)	376 (57.4)	1,020 (59.1)	−0.035	0.002
Medicaid, *n* (%)	94 (14.4)	211 (12.2)	0.062	0.014

SMD, standardized mean difference; adequate balance defined as SMD < 0.1 (all 17 covariates balanced after IPTW). ^*^Baseline GCS total = sum of eye, verbal, and motor components from the earliest synchronized assessment; complete-case for GCS. IPTW, inverse probability of treatment weighting; GCS, Glasgow Coma Scale; COPD, chronic obstructive pulmonary disease.

### Primary outcome: in-hospital mortality

**Figure 3:**
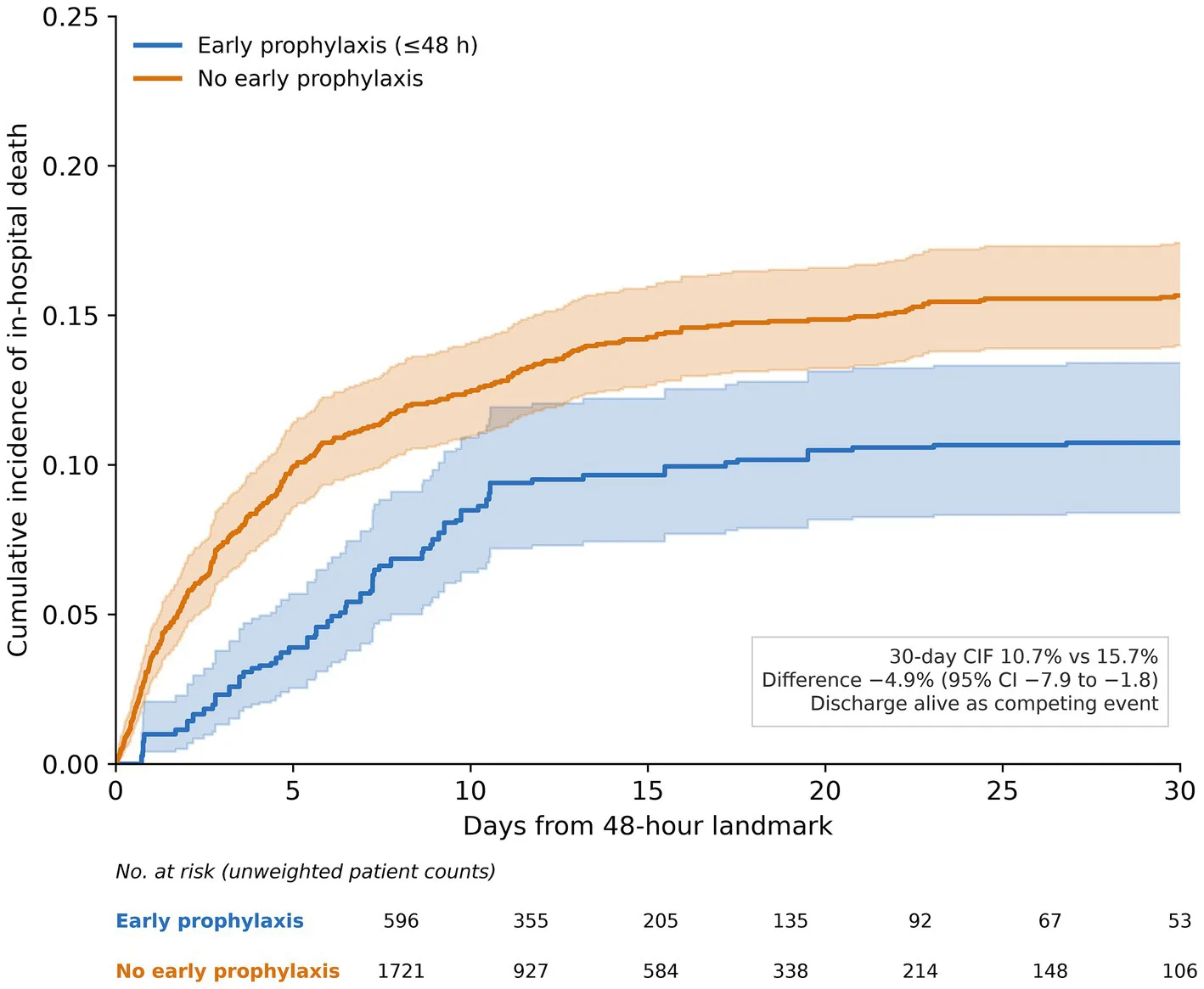
Weighted cumulative incidence of in-hospital death. Complete-case IPTW-weighted cumulative-incidence (Aalen–Johansen) curves from the 48-h landmark, with discharge alive as the competing event (complete-case *n* = 2,317). The 30-day cumulative incidence was 10.7% (95% CI 8.4–13.4) with early prophylaxis versus 15.7% (95% CI 14.0–17.4); difference −4.9% (bootstrap 95% CI −7.9 to −1.8). Numbers at risk are unweighted patient counts. The primary cumulative-risk estimate (OR 0.72, 95% CI 0.55–0.94; risk difference −4.0%) is reported in the text.

In-hospital death occurred in 371 patients: 71/655 (10.8%) with early prophylaxis versus 300/1,725 (17.4%) with no early prophylaxis. After IPTW, early prophylaxis was associated with lower in-hospital mortality: the primary estimate, treating discharge alive as the competing event, was an adjusted OR of 0.72 (95% CI 0.55–0.94; *p* = 0.017) with a weighted absolute risk difference of −4.0% (Table 2). A supporting weighted cumulative-incidence (Aalen–Johansen) analysis (complete-case, *n* = 2,317) was concordant, with a lower 30-day cumulative incidence of in-hospital death in the early group (10.7, 95% CI 8.4–13.4, vs. 15.7, 95% CI 14.0–17.4, difference −4.9%, bootstrap 95% CI −7.9 to −1.8; Figure 3). The supplementary cause-specific Cox model (discharge alive censored) gave an HR of 0.65 (95% CI 0.50–0.86, *p* = 0.003; Supplementary Figure 3); the unadjusted cause-specific HR was 0.55 (95% CI 0.43–0.72), and adjustment for measured covariates including GCS moved the estimate toward the null.

**Table 2:** Primary and secondary outcomes (IPTW-adjusted).

Outcome	Early prophylaxis (*n* = 655)	No early prophylaxis (*n* = 1,725)	Effect estimate (95% CI)	*p*
In-hospital death, *n* (%)	71 (10.8)	300 (17.4)		
Primary: adjusted OR			0.72 (0.55–0.94)	0.017
Weighted risk difference			−4.0%	–
Supporting: Aalen–Johansen 30-day CIF difference			−4.9% (−7.9 to −1.8)	–
Supplementary: cause-specific HR			0.65 (0.50–0.86)	0.003
Coded VTE, *n* (%)	53 (8.1)	109 (6.3)		
Adjusted OR			1.20 (0.85–1.69)	0.31

OR, odds ratio; HR, hazard ratio; CIF, cumulative incidence function; VTE, venous thromboembolism; CI, confidence interval. Primary in-hospital mortality estimate = weighted OR and risk difference (discharge alive not censored). The cause-specific HR censors discharge alive and is supplementary; coded VTE is a binary in-hospital outcome (no statistically significant difference).

### Secondary outcome: coded VTE

Coded VTE occurred in 162 patients (early prophylaxis 8.1%, no early prophylaxis 6.3%). After IPTW there was no statistically significant difference in coded VTE (adjusted OR 1.20, 95% CI 0.85–1.69; *p* = 0.31; unadjusted OR 1.31, 95% CI 0.93–1.84; Table 2).

### Sensitivity analyses

**Figure 4:**
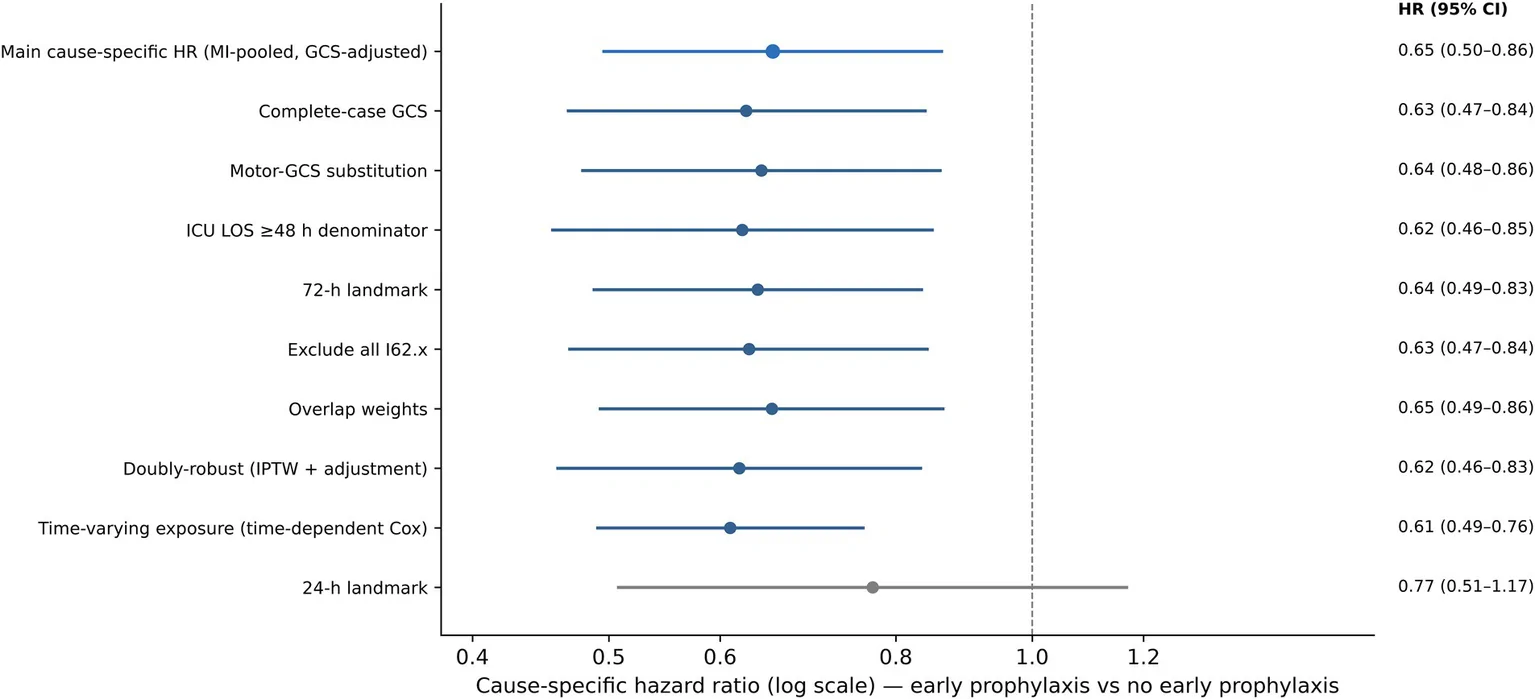
Sensitivity analyses for in-hospital mortality. Cause-specific hazard ratios (95% CI) for early prophylaxis versus no early prophylaxis, displayed in the same order in which the analyses are described in the text. The 24-, 48-, and 72-h landmark analyses each use their own landmark denominator (patients alive and in hospital at that window). The primary estimand for in-hospital mortality is the weighted odds ratio (0.72, 95% CI 0.55–0.94; risk difference −4.0%) reported in the text and Table 2; the hazard ratios shown here are the supplementary time-to-event estimates. The 24-h analyses was directionally consistent but not statistically significant (HR 0.77, 95% CI 0.51–1.17); all other analyses were statistically significant.

The mortality association was directionally consistent across most sensitivity analyses, although the 24-h estimate was not statistically significant (Figure 4): complete-case GCS (HR 0.63, 95% CI 0.47–0.84), motor-GCS substitution (0.64, 0.48–0.86), an ICU length-of-stay ≥48-h denominator (0.62, 0.46–0.85), a 72-h landmark (0.64, 0.49–0.83), exclusion of all I62.x codes (0.63, 0.47–0.84), overlap weighting (0.65, 0.49–0.86), and a doubly-robust model (0.62, 0.46–0.83). A model that treated prophylaxis initiation as a time-varying exposure, which reduces immortal-time bias, yielded HR 0.61 (95% CI 0.49–0.76). A 24-h landmark was directionally consistent but not statistically significant (HR 0.77, 95% CI 0.51–1.17), with fewer early-exposed patients. The E-value was 2.43 for the point estimate and 1.59 for the confidence limit nearest the null, indicating only limited robustness: an unmeasured confounder of moderate strength could account for the association.

### Subgroups and three-group description

In subgroup analyses, the association was similar with pre-landmark neurosurgery (HR 0.61, 95% CI 0.26–1.43; *n* = 325) and conservative management (HR 0.63, 95% CI 0.47–0.86; *n* = 1,992), with no evidence of effect modification (interaction *p* = 0.97 complete-case; *p* = 0.81 multiply-imputed). In an exploratory three-group description, unadjusted in-hospital mortality was 11.0% (early prophylaxis), 12.3% (delayed prophylaxis), and 20.2% (no prophylaxis; Supplementary Figure 2); the no-prophylaxis group showed markers consistent with greater clinical complexity and treatment-selection differences (higher intubation and neurosurgery frequency). This description is not an unbiased fixed-group effect comparison and the delayed-prophylaxis group carries residual immortal-time bias.

## Discussion

In this landmark cohort of 2,380 ICU patients with ICH, initiation of pharmacologic VTE prophylaxis within 48 h was associated with lower in-hospital mortality (primary weighted odds ratio 0.72, risk difference −4.0%; supporting Aalen–Johansen cumulative-incidence difference −4.9%; supplementary cause-specific hazard ratio 0.65) without a statistically significant difference in coded VTE. By classifying exposure at a 48-h landmark and including a baseline severity measure (GCS) in the propensity model, we addressed two limitations of earlier analyses; the association was directionally consistent across most sensitivity analyses, including a model that treated prophylaxis initiation as a time-varying exposure and reduced immortal-time bias, although the 24-h estimate was not statistically significant.

### Relationship to our earlier analysis and to prior work

We wish to be explicit about how this analysis differs from an earlier version of this work. That version excluded delayed initiators from the comparison and did not fully align exposure classification with the landmark, leaving residual immortal-time bias, and it did not adjust for neurological severity. Correcting these issues—placing delayed initiators in the comparator, fixing exposure at the landmark, and adjusting for GCS—attenuated the mortality association from a hazard ratio of approximately 0.41–0.65. The revised estimate is more credible and is what we report here. Our findings remain broadly consistent with observational literature and meta-analyses suggesting potential benefit of earlier thromboprophylaxis once imaging stability is documented (13, 24–26), while underscoring how strongly design choices influence the apparent magnitude of effect.

### Comparison with prior studies

Our results are broadly concordant with this body of work but differ in design in ways that matter. Early observational cohorts and a randomized trial of enoxaparin reported no clear excess of hematoma expansion when prophylaxis was begun after initial imaging stability (7–9), and systematic reviews and meta-analyses have similarly found that heparin-based thromboprophylaxis reduces VTE without a statistically significant increase in hematoma expansion or mortality, while cautioning that the evidence is of low certainty and the treatment windows are heterogeneous (10–13). Most of these studies either excluded delayed initiators or did not adjust for neurological severity; by retaining delayed initiators in the comparator and adjusting for baseline GCS, our landmark design produced a more attenuated and, we believe, more credible mortality association. Reported VTE rates also vary widely across ICH cohorts because of differences in surveillance and case ascertainment (4), consistent with our decision to treat coded VTE cautiously as an in-hospital binary outcome.

### Interpretation of the VTE result

The absence of a significant difference in coded VTE should be interpreted cautiously. VTE was ascertained from diagnosis codes without a reliable onset time, incident and pre-existing events cannot be fully separated in MIMIC-IV, and differential surveillance (longer-stay patients undergoing more imaging) may bias detection. We therefore report VTE only as coded in-hospital occurrence.

### Limitations

The principal limitation is residual confounding by indication. Although we adjusted for GCS, the strongest determinants of ICH prognosis and of treatment timing—hematoma volume, location, intraventricular extension, and hematoma expansion—are not available in MIMIC-IV. Clinicians plausibly delay prophylaxis in patients with larger or unstable hematomas and worse prognosis, so some or all of the observed association could reflect these unmeasured factors rather than an effect of prophylaxis; the three-group gradient (highest mortality in the no-prophylaxis group) is consistent with this. GCS itself was incompletely assessable (intubation), single-center data limit generalizability, hematoma expansion (a key safety endpoint) could not be evaluated, and goals-of-care and do-not-resuscitate status were unmeasured. For in-hospital mortality, discharge alive is a competing event; we therefore report the cumulative-risk estimands (odds ratio, risk difference, and Aalen–Johansen cumulative incidence) alongside a cause-specific Cox hazard ratio, which answer different questions but agreed in direction and magnitude here. The E-value indicates only limited robustness (2.43 for the point estimate; 1.59 at the confidence limit), and it does not offset the specific, known confounders that are absent from the database. These findings are association only and do not support a causal or safety conclusion.

### Clinical implications

The results are consistent with guidelines that permit initiation of pharmacologic prophylaxis within days of ICH in selected patients (1), but they cannot establish that early initiation causes lower mortality. Where pharmacologic prophylaxis is deferred because of bleeding concern, mechanical prophylaxis with intermittent pneumatic compression reduces deep vein thrombosis after stroke and is a reasonable adjunct (27). Decisions should remain individualized, weighing hematoma stability and bleeding risk. Randomized trials with standardized protocols and neuroimaging severity data are required.

## Conclusion

In this retrospective landmark cohort, initiation of pharmacologic VTE prophylaxis within 48 h of ICU admission was associated with lower in-hospital mortality, without a statistically significant difference in coded VTE, and directionally consistent across most sensitivity analyses, although the 24-h estimate was not statistically significant. Because hematoma volume, location, intraventricular extension, and expansion were unavailable, residual confounding by indication may explain part or all of the association; causality cannot be established. Randomized controlled trials are needed.

## Data Availability

Publicly available datasets were analyzed in this study. The data are available from the Medical Information Mart for Intensive Care IV (MIMIC-IV v3.1) database at https://physionet.org/content/mimiciv/3.1/. Access to MIMIC-IV requires completion of a data use agreement and human subjects research training (CITI Program). The analysis code is available from the corresponding authors upon reasonable request.

## Supplementary material

SUPPLEMENTARY FIGURE 1 Propensity-score overlap. Propensity-score distributions for early prophylaxis versus no early prophylaxis, showing common support (score range 0.055–0.716).

SUPPLEMENTARY FIGURE 2 Three-group descriptive outcomes. Unadjusted in-hospital mortality and coded VTE for the early-prophylaxis, delayed-prophylaxis, and no-prophylaxis groups. Descriptive only—not an unbiased fixed-group effect comparison; the delayed-prophylaxis group carries residual immortal-time bias. No between-group significance testing is presented.

SUPPLEMENTARY FIGURE 3 Cause-specific survival. Complete-case IPTW-weighted cause-specific Kaplan–Meier survival from the 48-h landmark, with discharge alive treated as censoring (complete-case *n* = 2,317); cause-specific hazard ratio 0.63 (95% CI 0.47–0.84). This time-to-event display is supplementary to the primary cumulative-incidence analysis (Figure 3), which treats discharge alive as a competing event.
